# Silencing of voltage-gated potassium channel K_V_9.3 inhibits proliferation in human colon and lung carcinoma cells

**DOI:** 10.18632/oncotarget.3517

**Published:** 2015-03-10

**Authors:** Jeong-Ha Lee, Jun-Won Park, Jun Kyu Byun, Hark Kyun Kim, Pan Dong Ryu, So Yeong Lee, Dae-Yong Kim

**Affiliations:** ^1^ Laboratory of Veterinary Pathology, Seoul National University, Seoul, Korea; ^2^ Biomolecular Function Research Branch, National Cancer Center, Goyang, Gyeonggi, Korea; ^3^ Veterinary Pharmacology, College of Veterinary Medicine and Research Institute for Veterinary Science, Seoul National University, Seoul, Korea

**Keywords:** cancer, colon, K_v_9.3, lung, proliferation

## Abstract

Voltage-gated potassium (K_v_) channels are known to be involved in cancer development and cancer cell proliferation. K_V_9.3, an electronically silent subunit, forms heterotetramers with K_V_2.1 in excitable cells and modulates its electrophysiological properties. However, the role of K_V_9.3 alone in non-excitable cancer cells has not been studied. Here, we evaluated the effect of silencing K_V_9.3 on cancer cell proliferation in HCT15 colon carcinoma cells and A549 lung adenocarcinoma cells. We confirmed the expression of K_V_9.3 mRNA in HCT15 and A549 cells and showed that silencing K_V_9.3 using small interfering RNA caused G0/G1 cell cycle arrest and alterations in cell cycle regulatory proteins in both HCT15 and A549 cells without affecting apoptosis. Also, stable knockdown of K_V_9.3 expression using short-hairpin RNA inhibited tumor growth in SCID mouse xenograft model. Using a bioinformatics approach, we identified Sp1 binding sites in the promoter region of the gene encoding K_V_9.3. We further found that Sp1 bound to this region and showed that the Sp1 inhibitor, mithramycin A, induced a concentration-dependent decrease in K_V_9.3 expression. Taken together, these data suggest that knockdown of K_V_9.3 inhibits proliferation in colon carcinoma and lung adenocarcinoma cell lines and may be regulated by Sp1.

## INTRODUCTION

Voltage-gated potassium (K_V_) channels are among the most diverse ion channel families [29]. In excitable cells, K_V_ channels regulate the membrane potential and contribute to the generation and propagation of action potentials [28]. In non-excitable cells, they are thought to be involved in regulating intracellular Ca^2+^ concentration, pH and cell volume, as well as cell-cycle progression, differentiation, and apoptosis [28, 29, 39]. Recent studies have also shown that K_V_ channels play an important role in cancer development and cell proliferation [22]. A variety of potassium channels, including the K_V_ channels K_V_1.1, K_V_1.3, K_V_4.1, K_V_10.1 and K_V_11.1, are thought to enhance cell proliferation [1, 12, 14, 19, 35]. However, how various K^+^ channels influence the cell cycle and cell proliferation remains elusive. The classic explanation is that K^+^ channels contribute to proliferation through permeation-related mechanisms that include membrane hyperpolarization, driving force generation for Ca^2+^, and cell volume regulation [35, 39]. However, recent studies have shown that non-conducting K^+^ channel mutants retain their proproliferative properties [3, 9, 26], suggesting that a non-conducting mechanism may be involved in K^+^ channel regulation of cell proliferation.

K_V_9.3 (*KCNS3*) is an electronically silent K_V_ α-subunit that does not form electronically functional channels when expressed as a homomultimer [30]. Silent K_V_9.3 α-subunits share substantial structural homology with K_V_2.1 channels and form heteromultimers with this latter subfamily, modulating their electrophysiological and pharmacological properties [30]. Compared with homomeric K_V_2.1 channels, heteromeric K_V_2.1/K_V_9.3 channels exhibit increased current amplitude, more rapid activation, and an altered steady-state activation shifted towards more negative values [30]. K_V_9.3, first cloned from rat pulmonary artery myocytes, is expressed most abundantly in the lung [30]. K_V_9.3 has also been detected in other tissues, including the rat brain, cerebral vascular smooth muscle, intestine, stomach, kidney and testes, and human urinary bladder smooth muscle, neurons, and the placental vasculature [6, 10, 37, 42]. There are also reports of K_V_9.3 expression in cancer cells [33, 34]. Although there are several reports on the role of K_V_9.3 in connection with K_V_2.1 in excitable cells, such as myocytes and neurons [6, 7, 18, 30, 42], the role of K_V_9.3 alone in cancer cells has not been studied.

In the present study, we investigated K_V_9.3 function in human HCT15 colon carcinoma and A549 lung carcinoma cells by specifically silencing K_V_9.3 using small interfering RNA (siRNA) and short-hairpin RNA (shRNA).

## RESULTS

### Expression of K_V_9.3 in HCT15 and A549 cells

**Figure 1:**
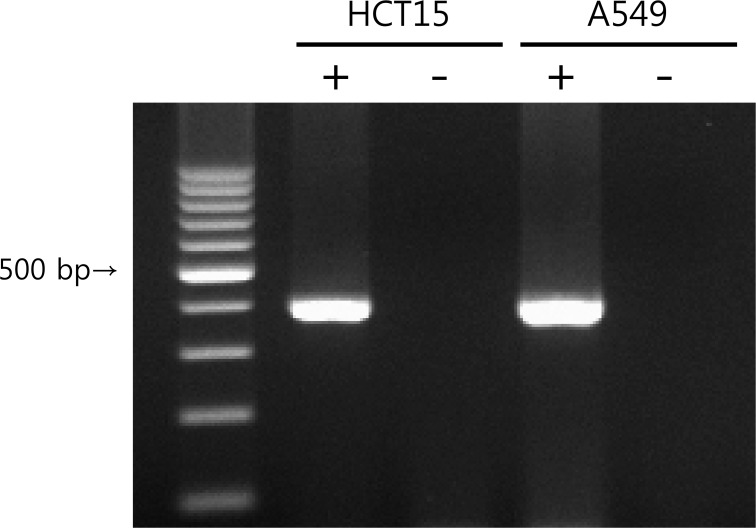
K9.3 mRNA expression in HCT15 and A549 cells PCR was performed using cDNA synthesized from total RNA isolates and the PCR products were run on 2% agarose gel. Negative controls without reverse transcriptase (−) were also made to confirm that there was no genomic DNA contamination.

K_V_9.3 mRNA expression was analyzed by RT-PCR. Using K_V_9.3-specific primers, we confirmed K_V_9.3 mRNA expression in HCT15 and A549 cells (Fig. 1). K_V_2.1 mRNA expression was also detected in both cell lines (Supplementary Fig. S1).

### siRNA-mediated K_V_9.3 knockdown decreases the viability of HCT15 and A549 cells

**Figure 2:**
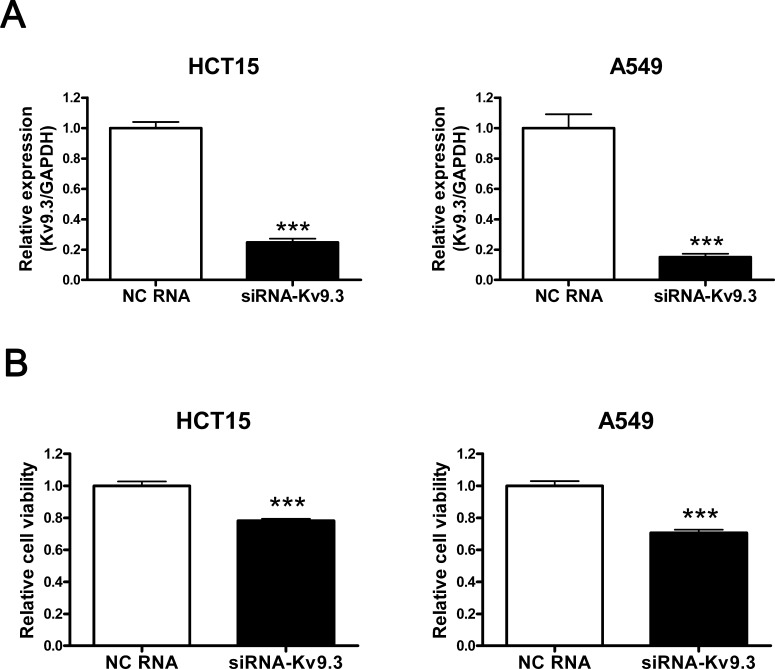
siRNA-K9.3 reduces cell viability of HCT15 and A549 cells A. Decreased expression of K_V_9.3 mRNA by K_V_9.3 siRNA treatment in HCT15 and A549 cells. The cells were harvested 48 h after K_V_9.3 siRNA or negative control RNA transfection. Real-time PCR was performed for 45 cycles to quantify the K_V_9.3 mRNA level. Each bar represents the mean ± S.E.M. (n=4, ***P < 0.001 by the Student's *t*-test versus negative control RNA treated group, NC: negative control) B. K_V_9.3 knockdown decreases cell viability of HCT15 and A549 cells. Cell viability was measured by MTT assay 72 h after K_V_9.3 siRNA transfection. Each bar represents the mean ± S.E.M. (n=9, three independent experiments, ***P < 0.001 by the Student's *t*-test versus negative control RNA treated group).

To examine the role of K_V_9.3 in cell viability, we transiently down-regulated K_V_9.3 using K_V_9.3 siRNA and performed MTT cell viability assays. siRNA treatment decreased K_V_9.3 mRNA levels by 75% (n = 4) and 85% (n = 4) in HCT15 and A549 cells, respectively (Fig. 2A). MTT assays revealed that K_V_9.3 siRNA treatment reduced the viability of HCT15 and A549 cells by 22% and 29%, respectively (n = 9, three independent experiments), compared to treatment with negative control siRNA (Fig. 2B). siRNA-mediated K_V_9.3 knockdown did not affect K_V_2.1 mRNA expression levels in HCT15 or A549 cell lines (Supplementary Fig. S2).

### siRNA-mediated K_V_9.3 knockdown induces G0/G1 cell cycle arrest in HCT15 and A549 cells but has no effect on apoptosis

**Figure 3:**
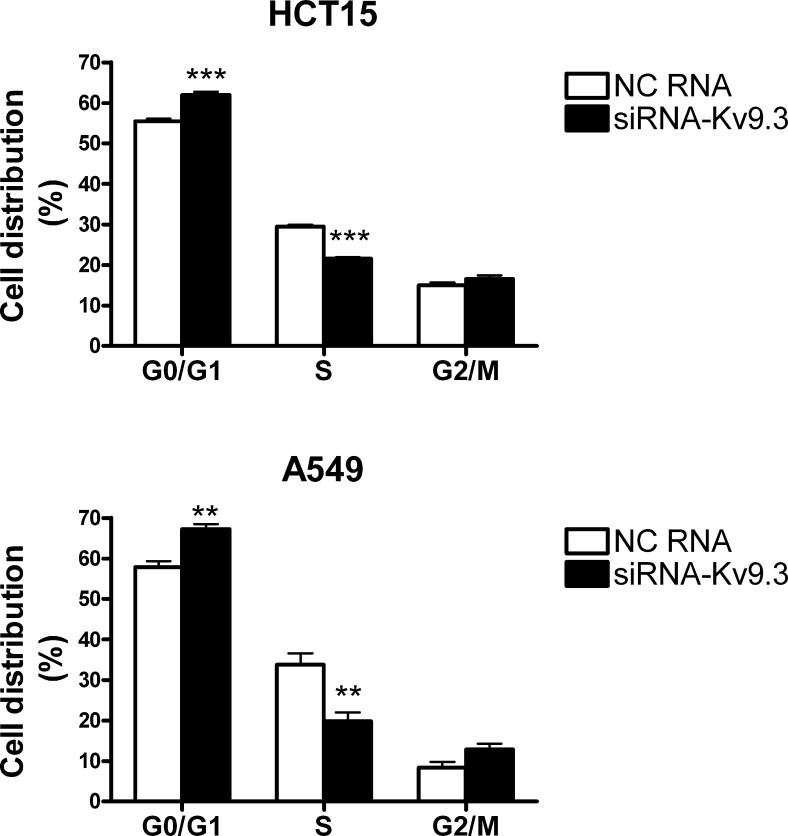
K9.3 knockdown induces alteration of cell cycle in HCT15 and A549 cells Cell cycle was analyzed by flow cytometry 72 h after K_V_9.3 siRNA transfection. Each bar represents the mean ± S.E.M. (n=4, three independent experiments, **P < 0.01, ***P < 0.001 by the Student's *t*-test versus negative control RNA treated group, NC: negative control).

To investigate whether the decrease in cell viability induced by K_V_9.3 siRNA treatment was attributable to alterations in the cell cycle or induction of apoptosis, we performed cell cycle and apoptosis analyses using flow cytometry. Fig. 3 shows that treatment with K_V_9.3 siRNA altered the distribution of cell cycle phases in both cell lines. K_V_9.3 knockdown increased the proportion of G0/G1-phase cells from 55.5% to 62.0% in HCT15 and from 57.9% to 67.3% in A549 cells. This increase was associated with a corresponding reduction in S-phase cells; the percentage of cells in this population decreased from 29.5% to 21.6% in HCT15 cells, and from 33.7% to 19.8% in A549 cells. K_V_9.3 knockdown had no significant effect on the G2/M phase distribution.

**Figure 4:**
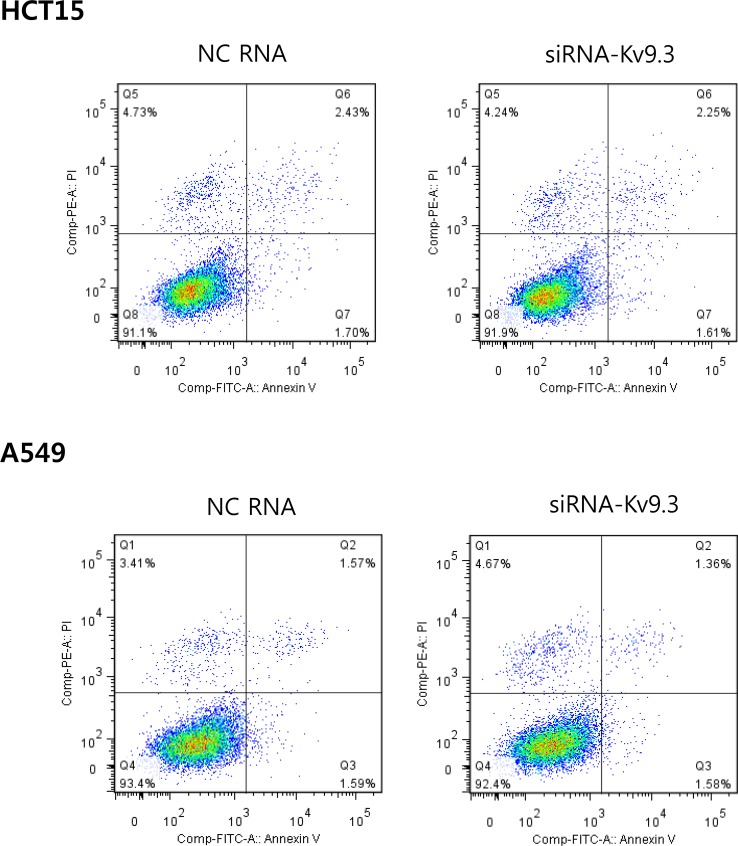
K9.3 knockdown does not have a significant effect on apoptosis in HCT15 and A549 cells After K_V_9.3 siRNA treatment (72 h), the cells were stained by annexin V and PI (double stain), and apoptosis was analyzed by flow cytometry. The percentage of cells in the lower right quadrant (annexin V positive, PI negative) was measured. No statistical difference was noted between the negative control RNA and K_V_9.3 siRNA treated group when compared by Student's *t*-test. (n=4, two independent experiments, NC: negative control).

An analysis of apoptosis using annexin V and PI double-staining revealed no difference in the percentage of apoptotic cells between K_V_9.3-knockdown and control groups (Fig. 4).

### siRNA-mediated K_V_9.3 knockdown modulates expression of cell cycle regulatory proteins related to G1-S phase transition in HCT15 and A549 cells

**Figure 5:**
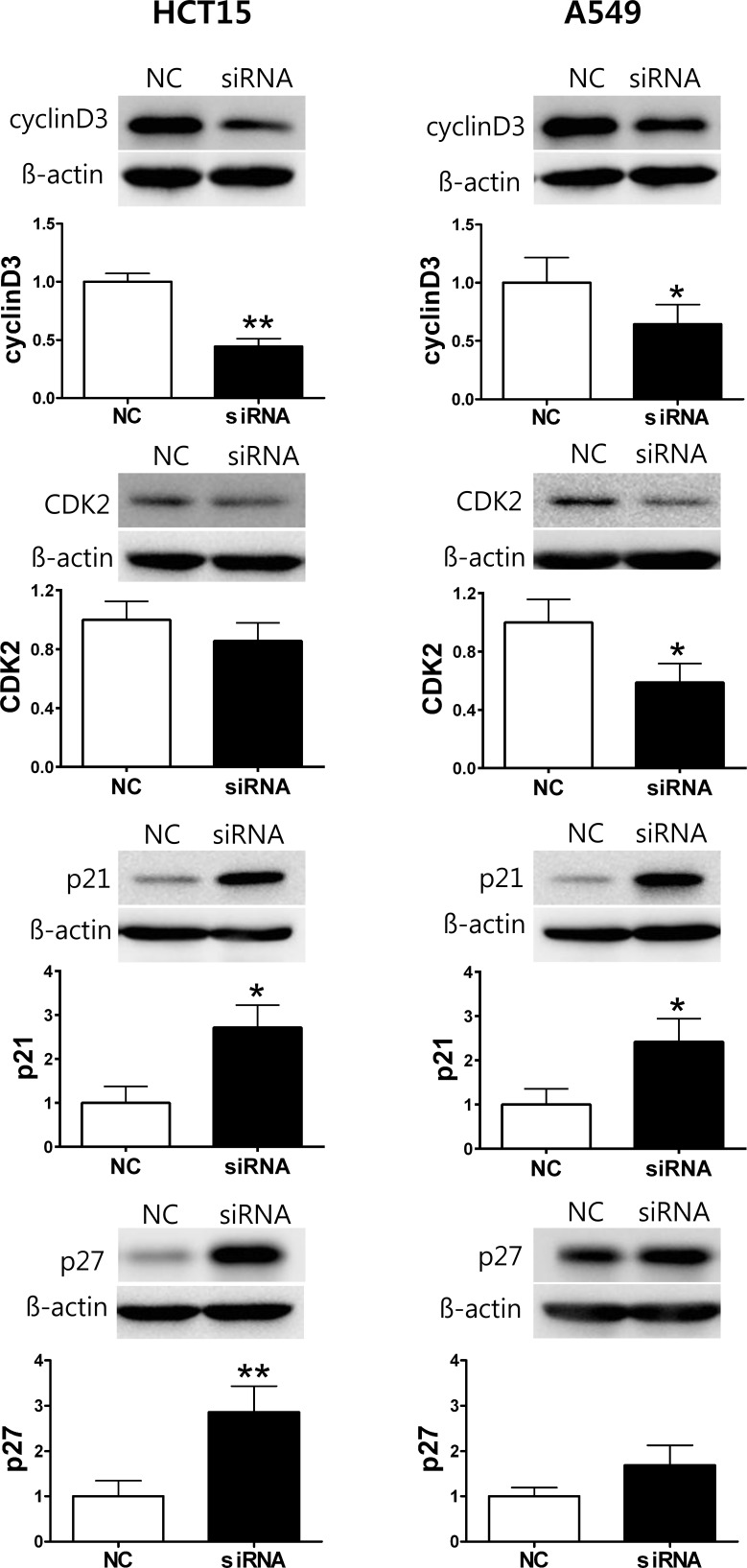
K9.3 knockdown changes protein expression level of cell cycle regulatory proteins in HCT15 and A549 cells The cells were harvested 72 h after K_V_9.3 siRNA transfection. Western blot was performed to examine expression of cyclin D3, CDK2, p21, p27. ß-actin was used as a loading control and, the protein expression level was normalized to that of the negative control RNA treatment group. Each bar represents the mean ± S.E.M. (n=3-5, *P < 0.05, **P < 0.01 by the paired Student's *t*-test versus negative control RNA treated group, NC: negative control RNA, siRNA: K_V_9.3 siRNA).

Next, we analyzed K_V_9.3 siRNA-induced changes in the expression of cell cycle regulatory proteins that participate in G1-S transition, examining the relative protein levels of cyclin D3, CDK2, p21, and p27. In HCT15 cells, K_V_9.3 knockdown significantly decreased cyclin D3 protein levels (0.44-fold relative to controls) and markedly increased p21 (2.7-fold) and p27 (2.9-fold) levels. CDK2 expression showed little change with K_V_9.3 knockdown in these cells. In A549 cells, K_V_9.3 knockdown decreased the expression of cyclin D3 and CDK2 protein (0.64-fold and 0.59-fold relative to controls, respectively) and increased the expression of p21 protein 2.41-fold. The expression level of p27 trended higher following K_V_9.3 knockdown, but this difference did not reach statistical significance (Fig. 5).

### Stable knockdown of K_V_9.3 using shRNA in HCT15 and A549 cells inhibits tumor growth *in vivo*

**Figure 6:**
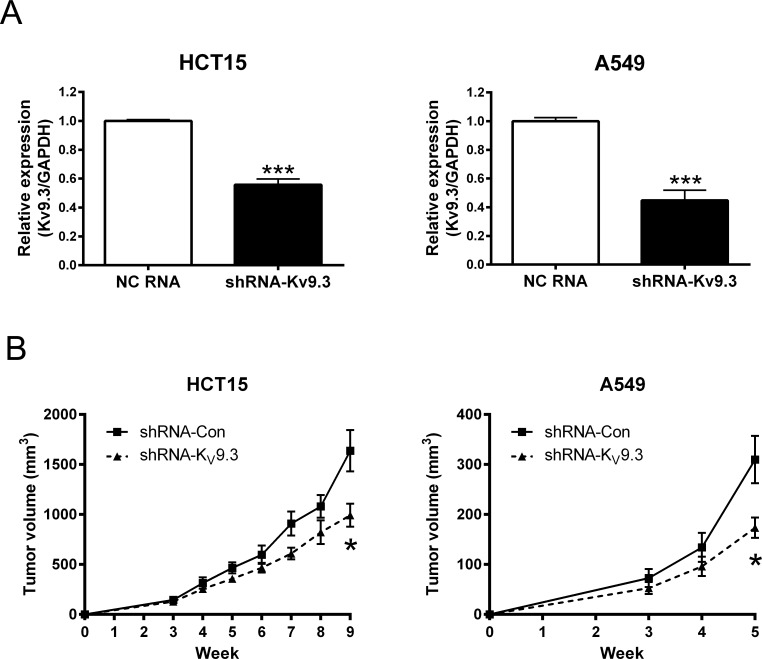
Stable knockdown of K9.3 using shRNA in HCT15 and A549 cells inhibits tumor growth *in vivo* A. Decreased expression of KV9.3 mRNA by K_V_9.3 shRNA treatment in HCT15 and A549 cells. Each bar represents the mean ± S.E.M. (n=4, ***P < 0.001 by the Student's *t*-test versus negative control shRNA treated group) B. Slower tumor growth *in vivo* of stable K_V_9.3 knockdown HCT15 and A549 cells. Each bar represents the mean ± S.E.M. (n=5, *P < 0.05 by the Student's *t*-test versus negative control shRNA treated group at that week).

To investigate whether silencing K_V_9.3 reduces tumor growth *in vivo*, we constructed mouse xenograft using stable K_V_9.3 knockdown HCT15 and A549 cell line. shRNA transfection decreased K_V_9.3 mRNA levels by 44% (n = 4) and 55% (n = 4) in HCT15 and A549 cells, respectively (Fig. 6A). In both cells, there was a tendency for stable K_V_9.3 knockdown cell lines exhibiting slower tumor growth *in vivo* compared to control cell lines. Statistical significance was noted on the 9^th^ week in HCT15 cells and on the 5^th^ week in A549 cells (n=5) (Fig. 6B).

### Inhibition of Sp1 by mithramycin A reduces K_V_9.3 transcription in HCT15 and A549 cells

**Figure 7:**
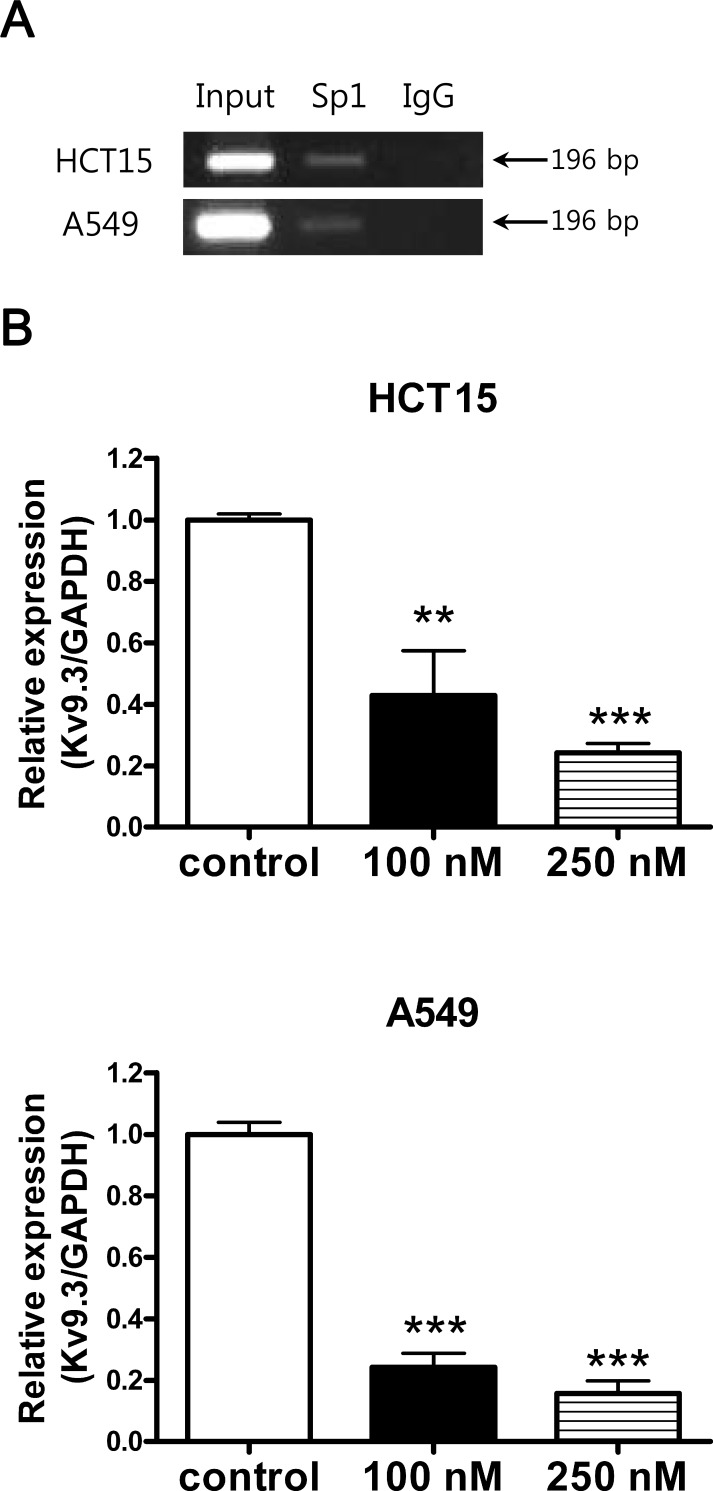
Inhibition of K9.3 gene expression by Sp1 inhibitor, mithramycin A A. Sp1 binds to K_V_9.3 promoter region. ChIP assay was performed with the anti-Sp1 or nonspecific rabbit (negative control) antibody. The GC rich region in the K_V_9.3 promoter region was amplified by RT-PCR. B. Inhibition of Sp1 by mithramycin A reduces K_v_9.3 expression in HCT15 and A549 cells. The cells were treated with mithramycin A (100 nM, 250 nM) for 24 h and K_V_9.3 mRNA expression level was measured by real-time PCR. Each bar represents the mean ± S.E.M. (n=4, two independent experiments, **P < 0.01, ***P < 0.001 by the Student's *t*-test versus control group).

We searched for possible transcription factor binding sites in the promoter region of the *KCNS3* gene encoding K_V_9.3 using the TFSEARCH program and found several possible Sp1 binding sites (G-C rich regions). To determine if Sp1 binds to the promoter region of *KCNS3*, we performed ChIP assay using a Sp1 antibody. These assays revealed that Sp1 bound to a predicted G-C rich binding site in both cell lines (Fig. 7A). To assess the functional consequences of this Sp1 binding, we tested whether inhibition of Sp1 with mithramycin A affected the expression of K_V_9.3. In both cells lines, mithramycin A significantly reduced the expression level of K_V_9.3 in a concentration-dependent manner (Fig. 7B). In HCT15 cells, 100 and 250 nM mithramycin A decreased K_V_9.3 expression by 0.43-fold and 0.24-fold relative to that in controls, respectively, whereas in A549 cells, the corresponding values were 0.24-fold and 0.16-fold (n = 4, two independent experiments).

## DISCUSSION

The role of K_V_9.3 has been investigated in excitable cells in conjunction with K_V_2.1 [6, 7, 16-18, 30, 42]. However, it has not been studied in non-excitable cancer cells in the context of proliferation. In this study, we focused on the effect of K_V_9.3 on proliferation in HCT15 and A549 cells, demonstrating that (i) K_V_9.3 transcripts are expressed in HCT15 and A549 cells; (ii) K_V_9.3 knockdown decreases the viability of HCT15 and A549 cells by inducing cell cycle arrest in G0/G1 phase with no change in the proportion of apoptotic cells; (iii) stable K_V_9.3 knockdown HCT15 and A549 cells show decreased tumor growth in mouse xenograft; and (iv) the transcription factor Sp1 potentially regulates the expression of K_V_9.3.

We confirmed that K_V_9.3 mRNA is expressed in HCT15 and A549 cells. Similarly, previous reports detected K_V_9.3 mRNA in colon cancer and uterine cancer cells [33, 34]. Unfortunately, the unavailability of a commercial K_V_9.3 antibody suitable for Western blot applications prevented us from confirming K_V_9.3 protein expression in HCT15 and A549 cells. In addition, we provided the first demonstration that silencing K_V_9.3 using siRNA exerted an anti-proliferative effect in HCT15 and A549 cells. To the best of our knowledge, all previous reports regarding the role of K_V_9.3 were related to the properties of K_V_9.3 assembling into heterotetrameric channels with K_V_2.1 and modulating its electrophysiological properties [6, 7, 10, 16, 18, 30, 42]. These properties of K_V_9.3 have been demonstrated in excitable cells and linked to several pathological and physiological processes, including pulmonary vasoconstriction in rat pulmonary artery myocytes [30], vasoconstriction in rat cerebral artery [42], and γ-oscillations in parvalbumin-containing neurons of schizophrenia patients [6]. In particular, K_V_2.1/K_V_9.3 heterotetramers are inhibited by hypoxia, resulting in increased concentrations of intracellular Ca^2+^ and hypoxic pulmonary artery vasoconstriction, leading to redistribution of non-oxygenated blood to more ventilated parts of the lung [11, 30, 32]. A similar role has also been suggested in human placental tissue [5]. However, no previous study has addressed whether K_V_9.3 has functions independent of K_V_2.1 or plays a role in non-excitable cells. Unlike these previous studies, we examined the specific function of K_V_9.3 in cancer cell lines by examining the effects of selective K_V_9.3 knockdown.

In the present study, we found that K_V_9.3 knockdown reduced proliferation of HCT15 and A549 cells by inducing cell cycle arrest in G0/G1 phase accompanied by changes in expression of cell cycle regulatory proteins related to G1-S transition. Cyclin D3 forms a complex with and functions as a regulatory subunit of CDK4 or CDK6, whose activity is required for G1-S transition [15]. p21 and p27 belong to the Cip/Kip family, whose members inhibit a variety of cyclin/CDK complexes [15]. CDK2 forms a complex with cyclin E that is necessary for progression from G1 to S phase [15]. Besides showing K_V_9.3 knockdown reduced proliferation *in vitro*, we observed that knockdown of this gene reduced tumor growth *in vivo* model (SCID mouse xenograft model). This strengthens our result that silencing K_V_9.3 has anti-proliferative effect by proving it in two different systems. It is now widely accepted that various potassium channels are involved in cancer cell proliferation [29, 35, 36, 39]. Inhibition or silencing of several potassium channels have shown anti-proliferative effect *in vitro* as well as *in vivo* system, most of them accompanied by G0/G1 cell cycle arrest. Examples are ATP-sensitive potassium (K_ATP_) channels in breast cancer cells [27, 40], K_V_4.1 channels in human gastric cancer cell lines [19] and tumorigenic human mammary epithelial cells [12], K_V_1.3 channels in lung adenocarcinoma cells [13], and K_V_11.1 channels in neuroblastoma cells [41]. In line with the previous studies, our findings expand on these previous works by showing K_V_9.3 inhibits cancer cell proliferation *in vitro* and *in vivo*.

Activation of potassium channels has been thought to promote apoptosis by reducing cell volume and intracellular potassium concentration [36] and various potassium channels are known to be involved in apoptosis [36]; however, we found that silencing K_V_9.3 in HCT15 and A549 cells did not induce apoptosis. This absence of an effect on apoptosis is similar to that observed following inhibition or silencing of K_V_1.3 in A549 cells [13] and K_ATP_ channel in a breast cancer cell line [27].

A role for K_V_9.3 independent of K_V_2.1 has not been extensively investigated. Although K_V_2.1 and K_V_9.3 mRNA expression have been reported to decrease concurrently in parvalbumin-containing neurons of schizophrenia patients [6], other studies have indicated divergent patterns of K_V_2.1 and K_V_9.3 expression. For example, in an analysis of the expression of potassium channels in the human placental vasculature, Wareing et al. (2006) found that, whereas K_V_2.1 was expressed in only a portion of the samples, K_V_9.3 was expressed in all samples [37]. Moreover, in screens of K_V_ channels in uterine carcinoma cell lines performed by Suzuki et al. (2004), several cell lines (HT-3, MS-751, Ishikawa) were positive for K_V_9.3 but negative for K_V_2.1 [34]. These findings indicate that K_V_9.3 can be expressed without K_V_2.1, implying that K_V_9.3 might have an independent role that does not involve K_V_2.1. In the current study, we confirmed that K_V_2.1 expression was not changed by K_V_9.3 knockdown. Thus, our demonstration that selective knockdown of K_V_9.3 influences the proliferation of cancer cells may suggest an action of K_V_9.3 independent of its association with K_V_2.1. In addition, it has been reported that K_V_9.3 contains numerous putative phosphorylation sites, including those for protein kinase A, protein kinase C, calcium-calmodulin kinase, and tyrosine kinase [30], suggesting that K_V_9.3 could be involved in other cellular events, such as cell cycle progression, through K_V_2.1-independent, non-conducting mechanisms that could involve protein-protein interactions. These potential K_V_2.1-independent functions of K_V_9.3 warrant further investigation.

In our search for transcription factors that might regulate the expression of K_V_9.3, we identified several GC-boxes known to be binding sites for Sp1 in the promoter of the corresponding *KCNS3* gene. We further found that Sp1 bound to the *KCNS3* promoter and showed that inhibition of Sp1 by mithramycin A decreased K_V_9.3 expression, supporting a role for Sp1 in regulating the expression of the *KCNS3* gene. Sp1 is a transcription factor containing three C2H2-type zinc finger DNA-binding domains that bind to GC-rich nucleotide sequences [2, 38]. Although Sp1 was first was thought to regulate housekeeping genes and other TATA-less genes, it has become evident that Sp1 is involved in diverse cellular events, including cell proliferation and cell cycle arrest [2, 38]. In addition, recent studies have shown that Sp1 also regulates expression of gene encoding different ion channels [8, 20, 24, 31] including K_V_ channels; in particular, K_V_1.5 [4], K_V_4.3 [23], and K_V_7.5 [21] have been reported to be targets of Sp1. Our findings expand on these previous works and broaden our understanding of the regulation of K_V_9.3.

In conclusion, our results demonstrate that specific knockdown of K_V_9.3 decreased cell viability through G0/G1 cell cycle arrest and tumor growth *in vivo*, and implicate Sp1 in regulating the expression of K_V_9.3 in HCT15 and A549 cells. This is the first report investigating the regulation of K_V_9.3 expression and the role of K_V_9.3 in cancer cell proliferation. Our results also suggest that K_V_9.3 may have functions independent of K_V_2.1 in cancer cells. Although further studies are needed to elucidate the detailed mechanisms by which K_V_9.3 influences cancer cell proliferation, our findings suggest that K_V_9.3 might serve as a potential target in cancer therapy.

## MATERIALS AND METHODS

### Cell culture

Human HCT15 colon carcinoma and A549 lung adenocarcinoma cell lines were purchased from the Korean Cell Line Bank (Seoul, South Korea). The cells were cultured in RPMI-1640 medium (Gibco, Grand Island, NY, USA) supplemented with 10% fetal bovine serum (Gibco) and 1% antibiotics (Zell Shield, Minerva Biolabs, Berlin, Germany), and incubated at 37°C in a humidified atmosphere of 5% CO_2_ in air.

### RNA extraction, reverse transcription-polymerase chain reaction (RT-PCR), and quantitative real-time PCR

Total RNA was extracted from HCT15 and A549 cells with TRIzol reagent (Life Technologies, Paisley, UK) according to the manufacturer's instructions. For cDNA synthesis, 1 μg of total RNA was reversed transcribed using a QuantiTect Reverse Transcription Kit (Qiagen, Hilden, Germany). Negative controls without reverse transcriptase were also run to confirm the absence of contamination. PCR was performed with an i-StarMaster Mix PCR Kit (iNtRON Biotechnology Inc., Sungnam, South Korea) according to the manufacturer's instructions using the following thermocycling conditions: 94°C for 5 min, followed by 35 cycles at 94°C for 40 s (denaturation), 60°C (for K_V_9.3) or 65°C (for K_V_2.1) for 40 s (annealing), and 72°C for 1 min (extension), with a final extension at 72°C for 7 min.

Real-time PCR was performed with a Rotor-Gene Q system (Qiagen) using a Rotor-Gene SYBR Green PCR kit (Qiagen). After denaturing for 5 min at 95°C, cDNA was amplified using 45 cycles of 95°C for 10 s and 60°C for 30 s. The target gene was quantified using the manufacturer's software (Rotor-Gene Q Series software, Qiagen). The relative fold change in target mRNA, normalized to glyceraldehyde-3-phosphate dehydrogenase (GAPDH), was measured using the delta-delta CT method [25]. The primers used for RT-PCR and real-time PCR are presented in Table 1.

**Table 1:** Primer pairs for RT-PCR and real-time PCR

	Gene	Sequence	Product size (bp)
RT-PCR	K_v_9.3	F: 5′-CTGGGGAAGCTGCTTACTTG-3′ R: 5′-CAGATTTTCTTCCGGAGCTG-3′	395
	K_v_2.1	F: 5′-GAATGTCCGCCGCGTGGTCCA-3′ R: 5′-CTTGGCTCTCTCCAGAGCCTC-3′	451
Real-time PCR	K_v_9.3	F: 5′-CAGTGAGGATGCACCAGAGA-3′ R: 5′-TTGCTGTGCAATTCTCCAAG-3′	200
	K_v_2.1	F: 5′-CTGCCAAGATCCTTGCCATAA-3′ R: 5′-CCGAACTCATCGAGGCTCTG-3′	103
	GAPDH	F: 5′-GAGTCAACGGATTTGGTCG-3′ R: 5′-TGGAATCATATTGGAACATGTAAAC-3′	135

### siRNA transfection

HCT15 and A549 cells were seeded in 6-well plates or 100-mm dishes 1 d before transfection. The cells were washed once with warm phosphate-buffered saline (PBS), and the growth medium was replaced with OPTI-MEM medium (Gibco). Cells were transfected with K_V_9.3 siRNA (FlexiTube siRNA Hs_KCNS3_3; Qiagen) or negative control RNA (AllStars Negative Control siRNA; Qiagen) at a final concentration of 10 nM using Lipofectamine 2000 (Life Technologies, Paisley, UK). Six hours after transfection, the medium was replaced with growth medium. The cells were harvested 48 and 72 h after transfection for RNA and protein extraction, respectively.

### Cell viability assay

Cell viability was determined using MTT (3-(4,5-dimethythiazol-2-yl)-2,5-diphenyl tetrazolium bromide) assays as described by the manufacturer (Sigma Aldrich, St. Louis, MO, USA). Briefly, 72 h after seeding HCT15 and A549 cells in 12-well plates and transfecting with siRNA as described above, 110 μl of a 5 mg/ml MTT solution was added to cells in 1 ml of media, yielding a final MTT concentration of 0.5 mg/ml. After 3 h of incubation at 37°C, the MTT solution was removed and the purple MTT formazan crystals were dissolved by adding 500 μl of dimethyl sulfoxide (DMSO) and shaking for 20 min. The dissolved formazan crystals were transferred to a 96-well plate, and absorbance was measured at 570 nm.

### Analysis of the cell cycle and apoptosis by flow cytometry

For cell cycle analyses, HCT15 and A549 cells were seeded in 25-T flasks and transfected with siRNA as described above. After 72 h, the cells were pelleted, washed and resuspended in ice cold PBS, and fixed with −20°C ethanol at a final concentration of 70%. The cells were then washed once with cold PBS, resuspended in PBS containing 50 μg/ml RNase A (Intron), and incubated for 30 min at 37°C. After adding propidium iodide (Sigma Aldrich) at a final concentration of 40 μg/ml, fluorescence was measured by flow cytometry using a BD FACSCalibur system (BD Bioscience, San Jose, CA, USA) and analyzed with CellQuest3.3 (BD Bioscience) and Mod Fit LT 3.3 software (Verity Software House, Topsham, ME, USA).

For determination of apoptosis, cells were seeded in 6-well plates and transfected with siRNA as described above. After 72 h, the cells were processed using an Annexin V Apoptosis Detection Kit FITC (eBioscience, San Diego, CA, USA), according to the manufacturer's instructions. Cell-associated fluorescence was measured using a BD FACS Canto II system (BD Bioscience) and analyzed using FlowJo software (TreeStar, San Carlos, CA, USA).

### Western blot analysis

Protein was extracted from HCT15 and A549 cells using CytoBuster Protein Extraction Reagent (Novagen, Madison, WI, USA) following the manufacturer's instructions. Equal amounts of protein (30 μg) were separated on 10% sodium dodecyl sulfate (SDS)-polyacrylamide gels and transferred to a 0.2 μm nitrocellulose membrane (Whatman GmbH, Hahnestraße, Germany). The membranes were blocked by incubating with Tris-buffered saline-Tween 20 (TBST) containing 10% skim milk (Difco, Sparks, MD, USA) for 2 h at room temperature. The blocked membranes were probed with anti-cyclin D3 (1:2000, 2936; Cell Signaling Technology, Danvers, MA, USA), anti-CDK2 (1:1000, sc-163; Santa Cruz Biotechnology, Santa Cruz, CA, USA), anti-p21 (1:1000, sc-397; Santa Cruz Biotechnology) or anti-p27 (1:500, sc-528; Santa Cruz Biotechnology) antibody at 4°C overnight. Membranes were incubated with horseradish peroxidase-conjugated horse anti-mouse IgG antibody (Vector Laboratories Inc., Burlingame, CA, USA) or goat anti-rabbit IgG antibody (Millipore, Billerica, MA, USA) for 1.5 h at room temperature. β-actin (Cell Signaling Technology) was used as an internal control.

### Establishment of stable knock down cells using lentiviral shRNA

To stably knockdown *KCNS3* (K_V_9.3) gene of HCT15 and A549 cell lines, lentiviral vector-mediated short-hairpin RNA (shRNA) construct was purchased from Sigma-Aldrich (St. Louis, MO) with pLKO.1-puro eGFP control vector (Sigma, SHC005). The target set was generated from accession number NM_002252: CCGGCCTTACTTTAACATTAGGGATCTCGAGAT CCCTAATGTTAAAGTAAGGTTTTTG. Lentiviruses were produced by cotransfecting shRNA-expressing vector and pMD2.G and psPAX2 constructs (Addgene, Cambridge, MA) into 293T cells by using lipofectamine 2000 (Invitrogen). Viral supernatants were harvested 48 hours after transfection, filtered through a 0.45 μm filter, titered, and used to infect HCT15 and A549 cells with 10 μg/mL polybrene. Cells were treated by 0.5 μg/mL puromycin at 48 hours after viral transduction and were selected for 10 days. Knockdown efficiency was determined by quantitative real-time RT-PCR.

### Xenograft assay

HCT15 and A549 cells (1 × 10^6^ cells in 50 μl of serum-free RPMI) were mixed with equal volumes of Matrigel (BD Biosciences, Bedford, MA) and injected into the subcutaneous flank tissue of the nonobese diabetic/severe combined immunodeficiency (NOD/SCID) mice. The mice were monitored weekly for tumor volumes, using a caliper. Tumor volume (*V*) was calculated by use of the following equation: V = 1/2 × *a* × *b*^2^, where *a* and *b* are the longest and shortest diameters of the tumor mass (in millimeters), respectively.

### Mithramycin A treatment

HCT15 and A549 cells were seeded in 6-well plates 1 d before mithramycin A treatment and harvested 24 h after treatment. Mithramycin A (Sigma Aldrich) was dissolved in methanol at a concentration of 1 mM and stored at −20°C. Immediately before use, the drug was diluted with culture medium to a concentration of 100 or 250 nM.

### Chromatin immunoprecipitation assay

HCT15 and A549 cells were grown on a 15-cm plate until they reached 70-80% confluence. Chromatin immunoprecipitation assays were performed using a ChIP-IT Enzymatic Kit (Active Motif, Carlsbad, CA, USA) according to the manufacturer's instructions. Cell lysates were immunoprecipitated with anti-Sp1 antibody (2 μg) (39058; Active Motif) or normal rabbit IgG (2 μg, sc-2027; Santa Cruz Biotechnology). The putative Sp1 binding site (G-C rich region) in the *KCNS3* promoter was predicted by the TFSEARCH program (http://www.cbrc.jp/research/db/TFSEARCH.html).

Sequences spanning the −428 to −233 bp region of the *KCNS3* promoter were amplified by PCR using the primer pair 5′-GGGGGAGGTCATCTTTTTCC-3′ (forward) and 5′-CCAGACAGGCGGACAGAC-3′ (reverse) at an annealing temperature of 57°C.

### Statistical analysis

All graphs were generated with GraphPad Prism4 (version 4.0; GraphPad Software, San Diego, CA, USA). Statistical significance was determined using unpaired or paired Student's *t*-test. A P-value less than 0.05 was considered statistically significant.

## SUPPLEMENTARY MATERIALS FIGURES


