# Supplementary material for: Silencing of voltage-gated potassium channel KV9.3 inhibits proliferation in human colon and lung carcinoma cells
Source: Oncotarget. 2015 Mar 10;6(10):8132–43. doi: 10.18632/oncotarget.3517 (PMC4480740; doi:10.18632/oncotarget.3517)
Supplement: Supplementary file 1 [file oncotarget-06-8132-s001.pdf]

Silencing of voltage-gated potassium channel K_v9.3 inhibits proliferation in human colon and lung carcinoma cells

Supplementary Material

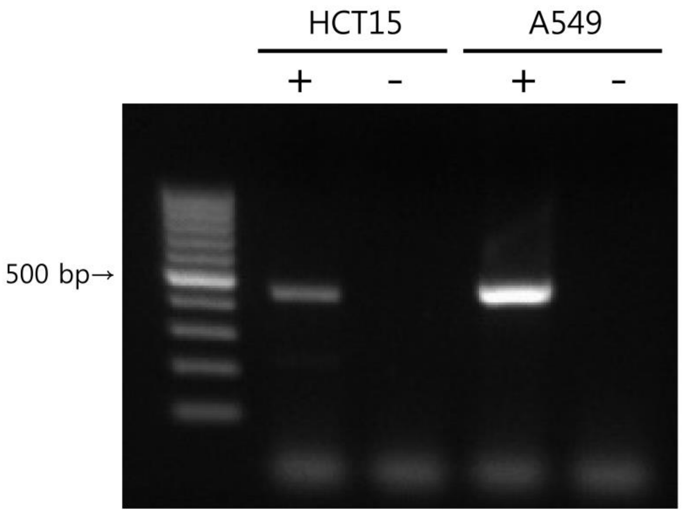

Figure S1: K_V2.1 mRNA expression in HCT15 and A549 cells. PCR was performed using cDNA synthesized from total RNA isolates and the PCR products were run on 2% agarose gel. Negative controls without reverse transcriptase (-) were also made to confirm that there was no genomic DNA contamination.

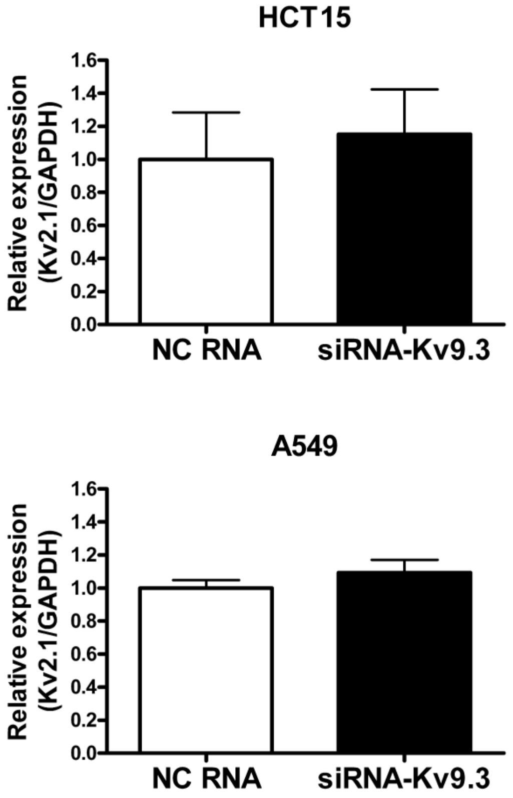

Figure S2: Expression of K_V2.1 mRNA after K_V9.3 siRNA treatment in HCT15 and A549 cells. The cells were harvested 48 h after K_V9.3 siRNA or negative control RNA transfection. Real-time PCR was performed for 45 cycles to quantify the K_V2.1 mRNA level. No statistical difference was noted between the negative control RNA and K_V9.3 siRNA treated group when compared by the Student's *t*-test. Each bar represents the mean ± S.E.M. (n=4, NC: negative control)
